# Accuracy of different Xpert MTB/Rif implementation strategies in programmatic settings at the regional referral hospitals in Uganda: Evidence for country wide roll out

**DOI:** 10.1371/journal.pone.0194741

**Published:** 2018-03-22

**Authors:** Winters Muttamba, Willy Ssengooba, Rogers Sekibira, Bruce Kirenga, Achilles Katamba, Moses Joloba

**Affiliations:** 1 Makerere University Lung Institute, College of Health Sciences, Makerere University, Kampala, Uganda; 2 Department of Medical Microbiology, College of Health Sciences, Makerere University, Kampala, Uganda; 3 School of Medicine, College of Health Sciences, Makerere University, Kampala, Uganda; Indian Institute of Technology Delhi, INDIA

## Abstract

**Background:**

Xpert MTB/RIF assay is a highly sensitive test for TB diagnosis, but still costly to most low-income countries. Several implementation strategies instead of frontline have been suggested; however with scarce data. We assessed accuracy of different Xpert MTB/RIF implementation strategies to inform national roll-out.

**Methods:**

This was a cross-sectional study of 1,924 adult presumptive TB patients in five regional referral hospitals of Uganda. Two sputum samples were collected, one for fluorescent microscopy (FM) and Xpert MTB/RIF examined at the study site laboratories. The second sample was sent to the Uganda Supra National TB reference laboratory for culture using both Lowenstein Jensen (LJ) and liquid culture (MGIT). We compared the sensitivities of FM, Xpert MTB/RIF and the incremental sensitivity of Xpert MTB/RIF among patients negative on FM using LJ and/or MGIT as a reference standard.

**Results:**

A total 1924 patients were enrolled of which 1596 (83%) patients had at least one laboratory result and 1083 respondents had a complete set of all the laboratory results. A total of 328 (30%) were TB positive on LJ and /or MGIT culture. The sensitivity of FM was n (%; 95% confidence interval) 246 (63.5%; 57.9–68.7) overall compared to 52 (55.4%; 44.1–66.3) among HIV positive individuals, while the sensitivity of Xpert MTB/RIF was 300 (76.2%; 71.7–80.7) and 69 (71.6%; 60.5–81.1) overall and among HIV positive individuals respectively. Overall incremental sensitivity of Xpert MTB/RIF was 60 (36.5%; 27.7–46.0) and 20 (41.7%; 25.5–59.2) among HIV positive individuals.

**Conclusion:**

Xpert MTB/RIF has a higher sensitivity than FM both in general population and HIV positive population. Xpert MTB/RIF offers a significant increase in terms of diagnostic sensitivity even when it is deployed selectively i.e. among smear negative presumptive TB patients. Our results support frontline use of Xpert MTB/RIF assay in high HIV/TB prevalent countries. In settings with limited access, mechanisms to refer smear negative sputum samples to Xpert MTB/RIF hubs are recommended.

## Introduction

Tuberculosis (TB) remains a global disease burden ranking alongside Human Immune deficiency virus (HIV) as the leading cause of death worldwide[1]. TB control efforts have been further complicated by the threat of Multi Drug Resistant Tuberculosis (MDR) and HIV. The 2016 WHO global TB report lists Uganda as a high HIV/TB country. In 2016, the prevalence of bacteriologically confirmed TB per 100,000 was 401 (292–509) while the incidence among all cases and HIV positive cases per 100,000 was 202 (120–304) and 66 (42–94) respectively. The same report indicates Uganda does GeneXpert (Xpert MTB/RIF, Cepheid, Sunnyvale, CA) testing for people at risk of HIV associated TB, people at risk of drug resistant TB and children as opposed to all TB presumptive patients [1]. The Xpert MTB/RIF assay is a simple to do test, however a national roll out of such an assay is dependent on operational and programmatic requirements which in most cases tends to be biggest challenge. Programmatic requirements would include a review or revision of available diagnostic algorithms, policies and guidance [2]. This particular study set out to assess the different Xpert MTB/RIF assay implementation strategies and this would greatly complement efforts in understanding the programmatic requirements that would guide country-wide roll out.

For more than 100 years, smear microscopy has been the backbone of TB diagnosis. Although this is inexpensive, it misses out on some cases due to its low sensitivity which gets even lower in certain scenarios like HIV co-infection [3–5]. The recent years have been dominated by advances in TB diagnostics all in the hope that TB diagnosis can be improved towards point of care (POC) tests. Such tests include a molecular-based Xpert MTB/RIF assay. The Xpert MTB/RIF assay was endorsed by WHO in 2011 as the initial diagnostic test in individuals suspected of having MDR-TB or HIV associated TB, with conditional recommendation for Xpert MTB/RIF to be considered as a follow-on test to microscopy in settings where MDR-TB or HIV is of lesser concern, especially in further testing of smear-negative specimens[6]. Xpert MTB/RIF is a rapid molecular diagnostic test that offers results of TB diagnosis as well as susceptibility to rifampicin within about 3 hours of sample receipt [7–9]. It has been hailed as a game changer and has been incorporated into many national TB control programs following large scale multi country evaluations [10]. Several studies done to find out the performance of Xpert MTB/RIF have found improved performance among the HIV positive population [11–13]. Low income, high TB burden countries continue to explore avenues for selective deployment of the Xpert MTB/RIF assay due to the high cost per test. A study done to assess the cost effectiveness of using Xpert MTB/RIF as an add on or replacement for smear microscopy found that both approaches are cost effective in low and middle-income countries [14]. This cost-effective study however didn’t take into consideration differences between HIV positive and negative individuals and effects of using one smear sample. It ignores the dropout rates associated with testing patients being asked to provide more than one sample on different days. Despite the WHO recommendations and the reduced cost of cartridges, the availability and deployment of the Xpert MTB/RIF as a point of care test will be slow due to the initial cost associated with procurement of the equipment and cartridges and difficulties resource limited settings experience in equipment calibration and maintenance [15].

The Xpert MTB/RIF assay has been found to have better sensitivity than smear microscopy [12, 13, 16–22]. Few studies have however evaluated the performance of this test in programmatic settings. This assay is very expensive and the cost is felt more by the low-income countries which also incur the biggest brunt of the high HIV and TB burden. There was thus a need for research to evaluate the use of Xpert MTB/RIF in Programmatic settings as well explore its role in clinical diagnostic algorithms. In line with a recommendation of a calculated and monitored roll out [14], a phased out roll out guided by programmatic evidence is key. The Ugandan health system is tailored for such a phased roll out due to the hierarchical nature of the health facilities starting from the referral hospitals which directly supervise the lower health facilities.

## Methods

### Study participants

This was a cross-sectional study of TB diagnostics among all TB presumptive adults attending regional referral hospitals in Uganda. The participants were both HIV positive and negative adults attending these study sites that were well spread across the country with some of the sites serving cross border populations by virtue of being near or in border towns. The participants had at least one cardinal symptom for TB. The study was conducted between October 2015 and August 2016.

To assess performance, two sputum samples were provided by each study participant i.e. spot and early morning. One sample was used for FM microscopy and Xpert MTB/RIF test at the study site while the other sample was sent to the central reference laboratory for Lowenstein Jensen (LJ) and Mycobacterial Growth Indicator Tube (MGIT) culture. HIV testing was done on the study participants as per the national HIV testing guidelines.

### Laboratory procedures

FM microscopy and Xpert MTB/RIF assay were done at the study sites according to standard procedures. Briefly smears were made from unprocessed sputum samples, stained using standard reagents and examined under direct Auramine O-stained FM (DFM) at ×100 and ×40 objectives (Olympus CX31 with LED attachment, Olympus Corporation, Tokyo, Japan). Smear results were reported as scanty, 1+, 2+ and 3+ using the WHO grading system[23]. The Xpert MTB/RIF assay was performed on the remainder of the sputum. Procedures for Xpert MTB/RIF were done using a 1:2 (sample: sample reagent) dilution. This was vigorously mixed and incubated at room temperature for 15 minutes and one mL of the mixture was transferred to the Xpert MTB/RIF cartridge. The cartridge was then inserted into the Xpert MTB/RIF machine; processing and result interpretation automated, using software version 4.0.

For laboratory procedures at the TB reference laboratory, the received sample was decontaminated using N-acetyl-L-cysteine (NALC)—Sodium hydroxide (NaOH; final concentration 1.5%) for 15 minutes at room temperature. Phosphate buffer saline (PBS; pH 6.8) was added up to 45 mL mark and centrifuged at 3,000g in a refrigerated centrifuge for 15 minutes. The supernatant were decanted and a pellet resuspended in 2 mL PBS, and vortexed. 0.5mL of the resuspended pellets were inoculated in MGIT and 3 drops each on two LJ tubes per sample for mycobacterial culture. Cultures on LJ were incubated at 37°c for up to 8 weeks and the MGIT in a machine for up to 6 weeks. Specimens positive for acid-fact bacilli (AFB) underwent Capillia Neo TB (TAUN, Numazu, Japan) testing. Capillia positive specimens were classified as *Mycobacterium tuberculosis* complex (MTB) and those negative as Non-tuberculous Mycobacteria (NTM). Those that were AFB-negative and had growth on blood agar were classified as contaminated and those without growth throughout were classified as negative.

### Statistical analysis

Data were double entered in an electronic database (Epidata Version 2.0 /2007), and where there were discrepancies, these were resolved by cross checking with the source documents (raw data). Data were exported to Stata v13 (Stata Corp, College Station TX, USA) for analysis. We used the exact binomial method for calculating 95% confidence intervals and the 2-sided Fisher’s exact test for comparing proportions.

Diagnostic yield was defined as the observed number of TB cases detected by each test employed while sensitivity per test was calculated as the proportion positive using MGIT and/or LJ culture as the reference comparator. The incremental sensitivity for an add-on strategy of Xpert MTB/RIF to smear microscopy was calculated as the number of participants positive by Xpert MTB/RIF but negative on the smear microscopy method divided by the total number of TB cases detected by MGIT. To calculate the incremental sensitivity of the Xpert MTB/RIF add-on strategy and LJ, we used MGIT as the reference comparator.

### Ethics statement

Approval was obtained from Makerere School of Public health IRB and national approval granted by Uganda National Council for Science and Technology (UNCST). All participants gave written informed consent.

## Results

### Characteristics of the study population

**Fig 1:**
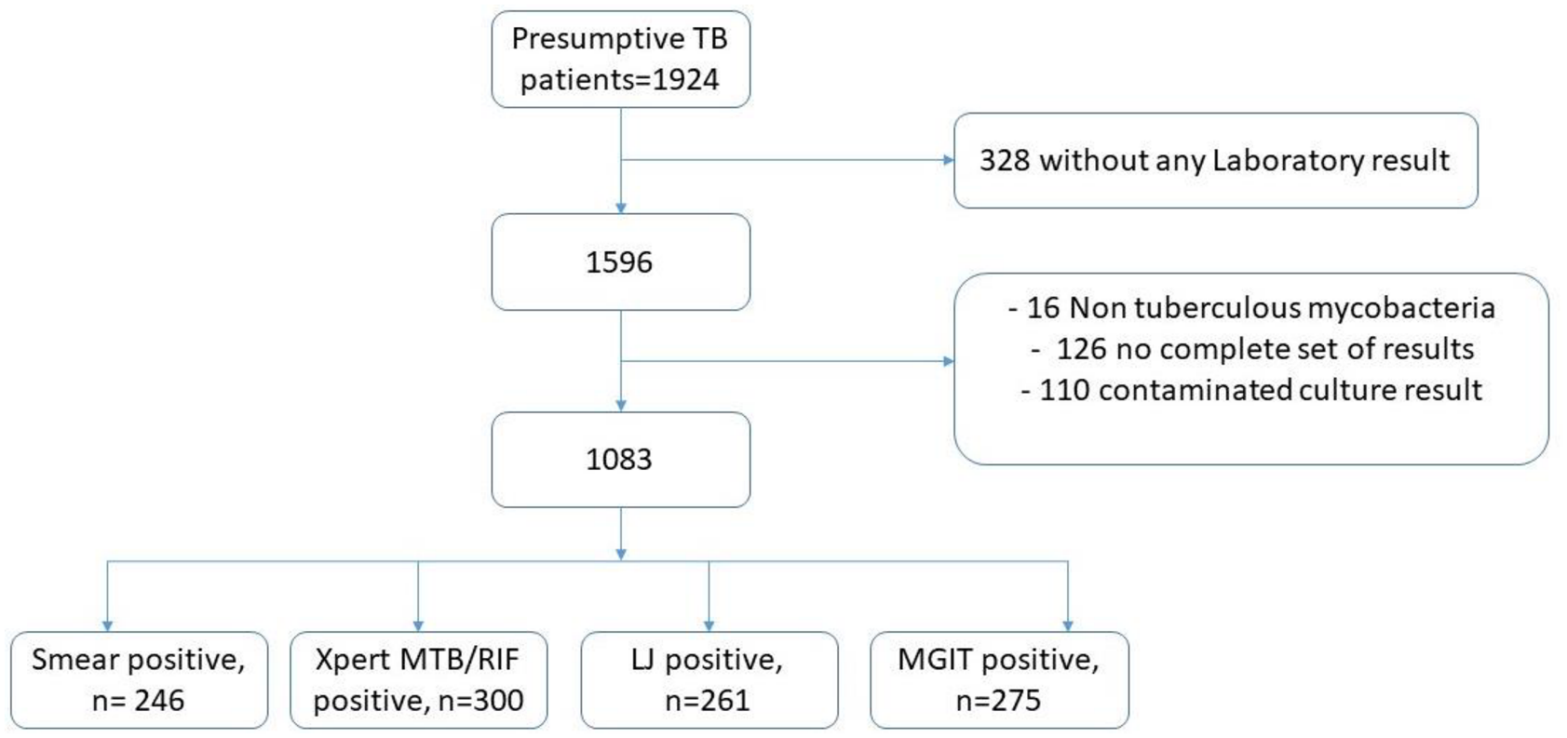
Flow chart of patients with laboratory complete results.

Of the 1924 eligible participants, only 1596 had at least one laboratory result of which 16 had non tuberculous mycobacteria (NTM) growth and 110 were contaminated on both cultures. Patients with either NTM or contaminated cultures were eliminated from the current analysis. A total of 1083 had a complete set of all the laboratory test results, Fig 1. Table 1 summarizes the demographic characteristics of the respondents that had all three sets of laboratory results. Majority of the respondents 869 (54.5%) were male. The age group of 18–38 years accounted for the majority 917 (57.4%) of the respondents and peasants 340 (21.3%) dominated the occupation group. More than half of the respondents 973 (58.6%) were married.

**Table 1:** Demographic characteristics of the study participants (n = 1,083).

Characteristic	Number	Percentage
*Age category*		
18-38yrs	917	57.4
39-59yrs	507	31.8
60+yrs	172	10.8
*Sex*		
Male	869	54.5
Female	727	45.5
*Occupation*		
Unemployed	249	15.6
Housewife	99	6.2
Peasant farmer	340	21.3
Market vendor	128	8.0
Builder	63	3.9
Health Worker	10	0.6
Business	300	18.8
Civil servant	61	3.8
Farmer	75	4.7
Other	267	16.8
*Education level*		
Tertiary	129	8.1
Secondary	546	34.3
Primary	729	45.9
None	186	11.7
*Marital status*		
Married	973	58.6
Unmarried	608	36.6
Unknown	80	4.8

### A combinational yield of different TB diagnostics

**Fig 2:**
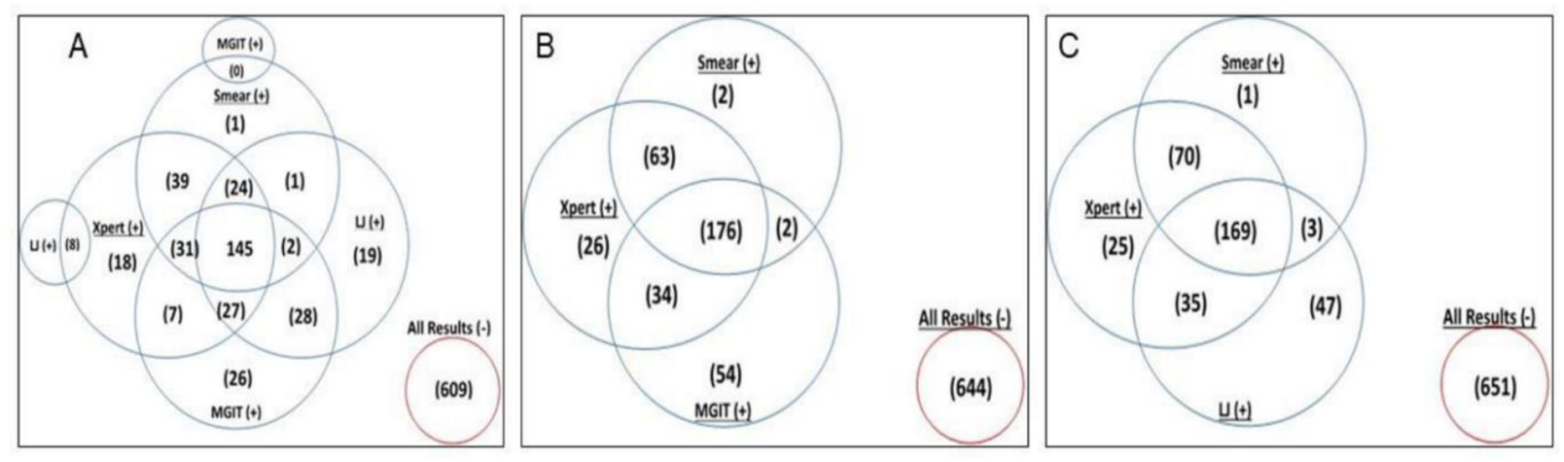
Venn diagram of yield per test and combinational yield of different TB diagnostic tests.

A total of 387 participants had at least one positive result by any of the methods. The Venn diagrams in Fig 2 show different combination of results obtained among smear, Xpert MTB/RIF, LJ and MGIT culture results. Panel A represents the yield from Xpert MTB/RIF and combinational yield from smear MGIT and LJ culture. Panel B represents yield from Xpert MTB/RIF and combinational yield from smear and MGIT culture, Panel C represents yield from Xpert MTB/RIF and combinational yield from smear and LJ culture.

### Performance of the different tests when MGIT and/or LJ is used as a reference test

The performance of the different tests when MGIT and/or LJ is used as a reference standard is represented in Table 2. When MGIT and LJ were combined as a gold standard, the sensitivity of MGIT was found highest across the different populations (general, HIV negative and HIV positive). followed by LJ, Xpert MTB/RIF and smear microscopy.

**Table 2:** Diagnostic accuracy of smear and Xpert MTB/RIF among the MGIT and/or LJ culture positive (n = 328) tuberculosis participants.

Test	General population			HIV-Negative			HIV-positive		
	Yield	Sensitivity, % (95%CI)	Specificity % (95%CI)	Yield	Sensitivity % (95%CI)	Specificity % (95%CI)	Yield	Sensitivity % (95%CI)	Specificity % (95%CI)
Smear	246	63.5 (57.9–68.7)	94.2 (92.2–95.8)	193	66.4 (59.9–72.4)	93.5 (91.1–95.4)	52	55.4 (44.1–66.3)	96.2 (91.6–98.6)
Xpert MTB/RIF	300	76.2 (71.7–80.7)	91.6 (89.3–93.6)	230	77.9 (72.1–83.1)	91.2 (88.5–93.5)	69	71.6 (60.5–81.1)	92.8(87.5–96.4)
LJ	261	79.6 (74.8–83.8)	100	194	81.2 (75.6–85.9)	100	63	75.9 (65.3–84.6)	100
MGIT	275	83.8 (79.4–87.7)	100	201	84.1 (78.8–88.5)	100	68	81.9 (71.9–89.5)	100

Key: LJ = Lowenstein Jensen, MGIT = Mycobacterium Growth Indicator Tube, CI = Confidence Interval HIV = Human Immunodeficiency Virus,

The sensitivity of all the tests dropped in the HIV positive population though the biggest drop was with smear microscopy 55.5% (44.1–66.3). The sensitivity of Xpert MTB/RIF is comparable in the general population and HIV negative population i.e. 76.2% (71.7–80.7) vs.77.9% (72.1–83.1) respectively while it drops a little in the HIV positive population; 71.6% (60.5–81.1).

The performance of the Xpert MTB/RIF and microscopy in the retreatment group wasn’t any different from the performance in the general population at 78.3% (56.3–92.5) and 66.7% (56.3–92.5) respectively.

### Incremental sensitivity of the possible Xpert MTB/RIF implementation strategies in the general and HIV positive populations

We analyzed data for the incremental sensitivities when Xpert MTB/RIF and LJ are used selectively in the general population and HIV positive populations. In the general population, Xpert MTB/RIF has an incremental sensitivity of 38.6% (28.4–49.6) while in the HIV positive population the incremental sensitivity is 40.0% (25.5–59.2). LJ increases the sensitivity in the FM negative Xpert MTB/RIF negative by 51.9% (37.8–65.7) and 50.0% (26.0–74.0) in the general and HIV positive population respectively.

**Fig 3:**
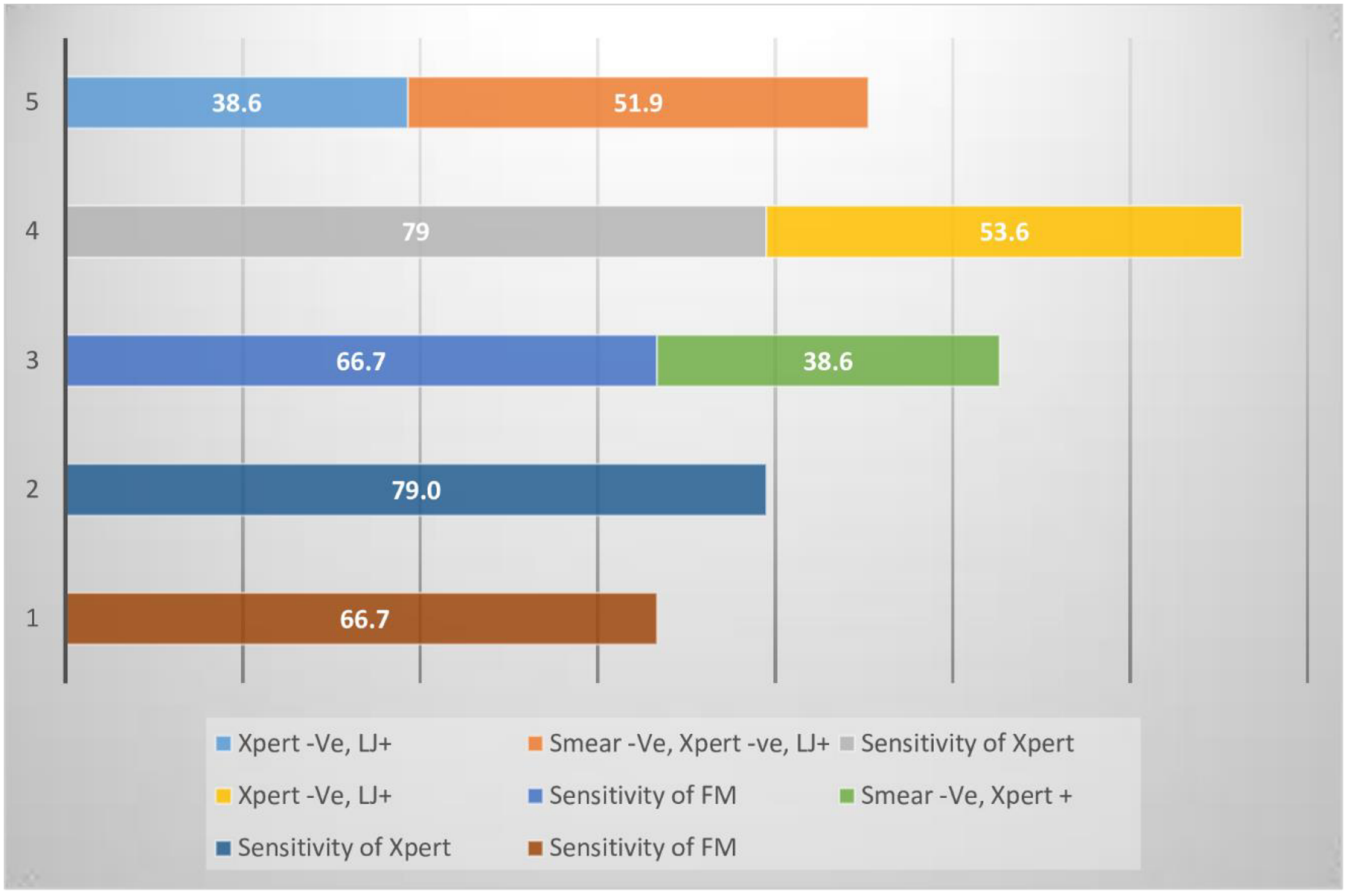
Incremental sensitivities when Xpert MTB/RIF and LJ are used selectively in the general population. Strategy Key: 1- Fronting Microscopy, 2- Fronting Xpert MTB/RIF, 3- Microscopy followed by Xpert MTB/RIF, 4- Xpert MTB/RIF followed by LJ Culture, 5- Microscopy, followed by Xpert MTB/RIF followed by LJ.

**Fig 4:**
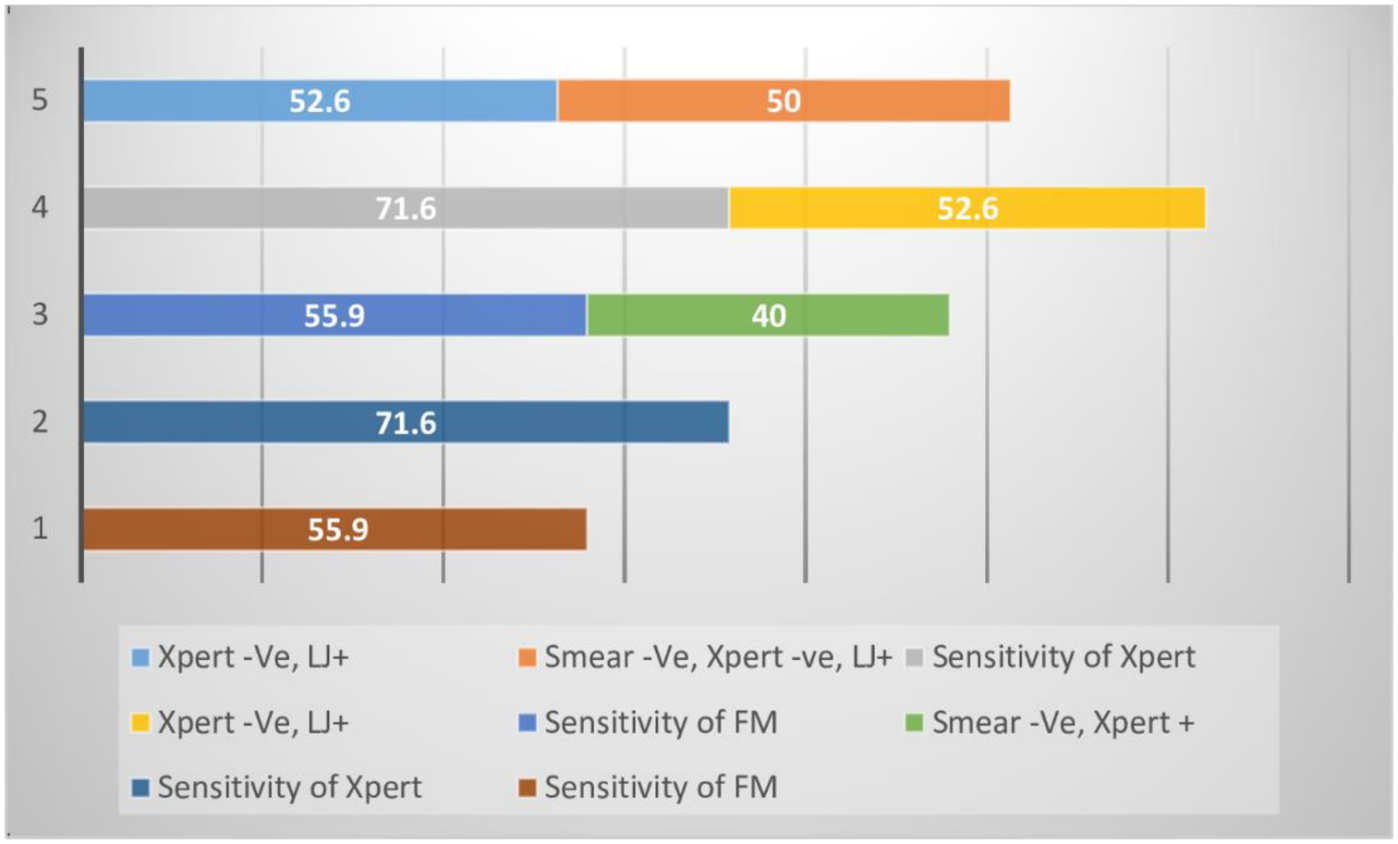
Incremental sensitivities when Xpert MTB/RIF and LJ are used selectively in the HIV positive population. Strategy: 1- Fronting Microscopy, 2- Fronting Xpert MTB/RIF, 3- Microscopy followed by Xpert MTB/RIF, 4- Xpert MTB/RIF followed by LJ Culture, 5- Microscopy, followed by Xpert MTB/RIF followed by LJ.

The two graphs also show there is added advantage in using the Strategy of fronting Xpert MTB/RIF and this advantage is further enhanced when LJ is added onto fronted Xpert MTB/RIF in both the general and HIV positive populations; Figs 3 and 4.

## Discussion

This study done at regional referral hospitals ascertains the performance of Xpert MTB/RIF assay under routine programmatic settings. We document the superior sensitivity of Xpert MTB/RIF over FM microscopy at regional referral hospitals.

There were more males than females among the respondents. This finding is similar to findings seen elsewhere which have found more males than females affected. The reasons for this are unclear but could include issues of accessibility to health services where women face more barriers to care than men [24]. Social culture differences could also be responsible for this difference due to reduced freedom for movement of women and over reliance on men for most matters including health and thus must negotiate several obstacles before accessing care [24]. The majority of the respondents were also in the age group of 18–38 years. This is the most vibrant and productive age group and this could thus have a significant impact on the economy.

This study reaffirms the findings of evaluation studies on performance of Xpert MTB/RIF done in controlled settings which showed better performance of Xpert MTB/RIF compared to microscopy. The results of this study are in agreement with results from previous studies done in other geographical areas and findings reaffirm the WHO recommendation for fronting Xpert MTB/RIF as the diagnostic test for tuberculosis based on its improved performance over microscopy.

The sensitivity of Xpert MTB/RIF in the general population and HIV positive population was higher than that of microscopy. This in agreement with studies done elsewhere that showed improved sensitivity of Xpert MTB/RIF [12, 16, 17]. As previously observed in other studies, [25–27], the sensitivity of microscopy is reduced in the HIV positive population. HIV infection limits the sensitivity of smear microscopy in diverse settings [25]. The reduced sensitivity of microscopy in HIV positive patients is explained by the paucibacillary nature of their TB and the fact that cavitating lesions are always missing in these patients [28]. This study establishes the impact of HIV infection on the overall diagnostic accuracy of the Xpert MTB/RIF. The sensitivity of Xpert MTB/RIF in the HIV positive population remained the same as in the general population. This is in agreement with findings from a study done in Tanzania and Peru [19, 29]. This is however different from two studies done which showed a reduced sensitivity of Xpert MTB/RIF in the HIV positive population [9, 17].

Much as this this particular study assessed the performance of Xpert MTB/RIF in adults and not children, the Xpert MTB/RIF assay has also been found to increase TB case detection in children [30–32].

The sensitivity of Xpert MTB/RIF as ascertained in this study is in agreement with findings obtained from a systemic review [12]. At 37.3%, the incremental yield obtained in this study is however lower than the findings from this review. It’s also lower than findings from a South African study which showed a sensitivity of 55% in the smear negative cases[17]. The difference could be a constellation of many factors including the nature of collected specimens (spot versus morning), geographical distribution of study sites which were distant from the reference laboratory and the sputum manipulation procedures done (processed versus un processed samples). The sensitivity obtained in this study is also lower than the findings from a study done in Thailand which showed a sensitivity of 81% among culture positive, smear negative patients [33]. The Thailand study was however done on a small sample size of 494 patients. The incremental sensitivity ascertained in this study is only 4% higher than findings from a recent study done in the region [34]. The study however had a smaller sample size compared to the current study and used the same sample for all the three tests unlike this one that used a different sample for culture. The incremental sensitivity among the HIV positive patients in this study (41.7%) was higher than the 35.7% found in the same study done in the region [34].

An additional 60 (7.7%) cases that had been missed by microscopy were detected by Xpert MTB/RIF. These ideally are patients that would miss out on TB treatment initiation if Xpert MTB/RIF wasn’t done. In a resource limited setting and where Xpert MTB/RIF is less affordable, well done fluorescent smear microscopy would detect 24% of the TB cases. However, there is need to critically assess the smear negative cases and where there is a sputum referral mechanism to a facility with Xpert MTB/RIF, further testing with this technology should be encouraged. In facilities that have Xpert MTB/RIF, it’s important to front this technology as using it as an add-on expends significantly more resources and time [35], yet the undiagnosed cases are known to contribute significantly to ongoing transmission in the community [36].

The sensitivity of Xpert MTB/RIF and microscopy in this study is lower than findings from a study done in another sub-Saharan country[13]. This could probably be due to the fact that this study didn’t use the same sample for all the tests unlike the South African study. This is also the possible reason why the sensitivity in the HIV group was lower than what was found in another South African study [11]. This study was done under routine programmatic settings where concentration techniques for sputum samples are not feasible. The sensitivity of Xpert MTB/RIF as ascertained in this study in the general population is comparable to findings from another study done in South Africa [17].

In terms of strategy, there are added benefits in using the Xpert MTB/RIF as an initial diagnostic test as opposed to using microscopy. This is largely because of the improved sensitivity of the Xpert MTB/RIF assay over microscopy. Though using Xpert MTB/RIF as an add-on to microscopy leads to better overall sensitivity, this is watered down by the risk of patients dropping out of the diagnostic cascade due to failure of clients to return to give a second sample. This however could be overcome by using the same sputum sample to run both tests i.e. microscopy and Xpert MTB/RIF. In settings where there are resource constraints and is very costly to front the Xpert MTB/RIF, this could be a worthwhile strategy.

In settings that aren’t resource constrained, fronting Xpert MTB/RIF would be ideal as this offers better sensitivity over microscopy and has the advantage of detecting Rifampicin resistant cases. This study done at regional referral hospitals puts forward a case for equipping the regional laboratories to be able to do LJ. This study shows the added advantage of using LJ in addition to Xpert MTB/RIF as an-add on test as it shows the incremental sensitivity of up to 50%. This would be one step towards decentralizing the laboratory services in the country and augurs well with the fact that these regional centers have since been transformed into MDR management centers. Bearing in mind that contamination is always a problem when these centers normally refer sputum to the central reference laboratory for culture and sensitivity, these centers can also be given capacity to prepare isolates which can then be shipped to the reference laboratory for drug sensitivity testing.

It was expected that the treatment status of the patient could affect the performance of the tuberculosis test. However this was not the case as the sensitivity of Xpert MTB/RIF and microscopy in the retreatment patients stayed the same as that in the general population. No previous studies have been done to ascertain if the sensitivity of tuberculosis diagnostics is altered by a patient’s previous treatment history. In this study, we were able to establish that the TB treatment history did not affect the performance of the fluorescence microscopy and Xpert MTB/RIF.

Our study had some limitations; we were unable to use the same sample across the cascade of tests but rather a different sample for culture and same sample for FM and Xpert MTB/RIF. This however didn’t affect the sensitivity of the tests. This effect however might have been resolved by the fact that the same sputum sample was used for both FM and Xpert MTB/RIF and the type of sample for culture was random.

## Conclusion

Xpert MTB/RIF has a higher sensitivity than fluorescent smear microscopy both in general and HIV positive populations at regional referral hospitals in Uganda. Xpert MTB/RIF offers a significant increase in terms of diagnostic sensitivity even when it is deployed selectively i.e. among smear negative presumptive TB patients. The increase is more marked in the HIV positive population. Our results support frontline use of Xpert MTB/RIF assay in high HIV/TB prevalent countries. In settings with limited access, mechanisms to refer smear negative sputum samples to Xpert MTB/RIF hubs are recommended. The added diagnostic benefit of LJ culture to Xpert MTB/RIF negative requires more studies.

## Supporting information

S1 File Dataset. (XLS)
